# Low dose of emetine as potential anti-SARS-CoV-2 virus therapy: preclinical in vitro inhibition and in vivo pharmacokinetic evidences

**DOI:** 10.1186/s43556-020-00018-9

**Published:** 2020-11-30

**Authors:** Aoli Wang, Yong Sun, Qingwang Liu, Hong Wu, Juan Liu, Jun He, Junling Yu, Qing Qing Chen, Yinglu Ge, Zhuhui Zhang, Chen Hu, Cheng Chen, Ziping Qi, Fengming Zou, Feiyang Liu, Jie Hu, Ming Zhao, Tao Huang, Beilei Wang, Li Wang, Wei Wang, Wenchao Wang, Tao Ren, Jing Liu, Yehuan Sun, Song Fan, Qibing Wu, Chaozhao Liang, Liangdan Sun, Bin Su, Wei Wei, Qingsong Liu

**Affiliations:** 1 Anhui Province Key Laboratory of Medical Physics and Technology, CAS Key Laboratory of High Magnetic Field and Ion Beam Physical Biology, Institute of Health and Medical Technology, Hefei Institutes of Physical Science, Chinese Academy of Sciences, Hefei, Anhui 230031 P. R. China; 2 Hefei Cancer Hospital, Chinese Academy of Sciences, Hefei, Anhui 230031 P. R. China; 3 Key Laboratory for Medical and Health of the 13th Five-Year Plan, Anhui Provincial Center for Disease Control and Prevention, Hefei, Anhui 230601 P. R. China; 4 Precision Targeted Therapy Discovery Center, Institute of Technology Innovation, Hefei Institutes of Physical Science, Chinese Academy of Sciences, Hefei, Anhui 230088 P. R. China; 5 Precision Medicine Research Laboratory of Anhui Province, Hefei, Anhui 230088 P. R. China; 6 Department of Epidemiology and Health Statistics, School of Public Health, Anhui Medical University, Hefei, China; 7 Department of Urology, The First Affiliated Hospital of Anhui Medical University, Institute of Urology, Anhui Medical University, Anhui Province Key Laboratory of Genitourinary Diseases, Hefei, Anhui China; 8 Department of Dermatology, The First Affiliated Hospital of Anhui Medical University, Hefei, China; 9 Key Laboratory of Dermatology (Anhui Medical University), Ministry of Education, Hefei, China; 10 Key Laboratory of Major Autoimmune Diseases, 218 Jixi Road, Hefei, 230022 Anhui China; 11 Institute of Clinical Pharmacology, Anhui Medical University, Key Laboratory of Anti-Inflammatory and Immune Medicine, Ministry of Education, Anhui Collaborative Innovation Center of Anti-Inflammatory and Immune Medicine, Hefei, 230032 China; 12 Institute of Physical Science and Information Technology, Anhui University, Hefei, Anhui 230601 P. R. China

**Keywords:** Emetine, COVID-19, Anti-SARS-CoV-2 infection therapy, Old drug repurposing

## Abstract

The global pandemic of COVID-19 has attracted extensive drug searching interets for the new coronavirus SARS-CoV-2. Although currently several of clinically used “old” drugs have been repurposed to this new disease for the urgent clinical investigation, there is still great demand for more effective therapies for the anti-infections. Here we report the discovery that an “old” drug Emetine could potently inhibit SARS-CoV-2 virus replication and displayed virus entry blocking effect in Vero cells at low dose. In addition, Emetine could significantly reduce the lipopolysaccharide (LPS) induced interleukin-6 (IL-6) protein level and moderately reduce the tumor necrosis factor (TNF-α) protein level in the M1 polarized THP-1 macrophages. In vivo animal pharmacokinetics (PK) study revealed that Emetine was enriched in the lung tissue and had a long retention time (over 12 h). With 1 mg/kg single oral dose, the effective concentration of Emetine in lung was up to 1.8 μM (mice) and 1.6 μM (rats) at 12 h, which is over 200-fold higher than the EC_50_ of the drug. The potent in vitro antiviral replication efficacy and the high enrichment in target tissue, combining with the well documented safety profiles in human indicate that low dose of Emetine might be a potentially effective anti-SARS-CoV-2 infection therapy.

**Supplementary Information:**

The online version contains supplementary material available at 10.1186/s43556-020-00018-9.

## Introduction

The worldwide pandemic of Coronavirus disease 2019 (COVID-19) caused by a novel coronavirus SARS-CoV-2 since December of 2019 has quickly emerged as a global public health crisis [1]. Based on the John Hopkins CSSE data (https://coronavirus.jhu.edu/map.html), until September 25th, 2020, more than 32 million confirmed cases were identified across over 188 countries and regions in the world and already led to more than 980 thousand deaths. SARS-CoV-2 is a single-stranded RNA virus belonging to the β-coronavirus (β-CoV) cluster with 79% nucleotide similarity to severe acute respiratory syndrome coronavirus (SARS-CoV) and 50% similarity to Middle East respiratory syndrome coronavirus (MERS-CoV) [2]. SARS-CoV-2 caused severe symptoms including fever, cough and shortness of breath that can lead to pneumonia and even death [3, 4]. In addition, the elevated expression level of inflammatory cytokines such as IL-6 and TNF-α etc. has been observed in the severe patients [5]. The high morbidity of SARS-CoV-2 infection and the lack of effective treatment currently brought an urgent demand for seeking for effective therapies. Although several drugs, such as Interferon, Ribavirin, Lopinavir-ritonavir, Remdesivir, Chloroquine, Hydroxychlroquine as well as some traditional Chinese medicines (TCM) exhibited primary efficacy either in vitro or in the clinical investigation for COVID-19 treatment (NCT04254874; NCT04276688; NCT04252885; NCT04252664; NCT04261517; NCT04303507; NCT04251871). Currently, there are still no effective drugs approved for the treatment of SARS-COV-2 infection yet. In addition, given the fact that a large portion of the infections accompanying with basic diseases such as diabetes, cancer, hypertension etc. [6], more antiviral therapies are still highly demanded.

Emetine, available either as a pure chemcial or in the form of Ipecac syrup, is known as an anti-protozoal drug for amoebiasis, vomiting inducing reagent as well as anti-cough reagent. It has been reported to show broad-spectrum antiviral activities including the HIV [7], Zika virus (ZIKV), Ebola virus (EBOV) [8], as well as Coronavirus such as SARS-CoV, MERS-CoV [9], Coronavirus mouse hepatitis virus A59 (MHV-A59), Human Coronavirus OC43 (HCoV-OC43) and Human Coronavirus NL63 (HCoV-NL63) [10]. The potent anti-coronavirus activity of Emetine indicates that it might also be a potential therapeutic agent combating SARS-CoV-2 [11, 12]. Thus, we investigated the in vitro antiviral activities of Emetine against SARS-COV-2 infection and further explored its in vivo pharmacokinetic profiles for proposing a clinical investigation translation.

## Results

### In vitro antiviral effect of emetine

**Fig. 1:**
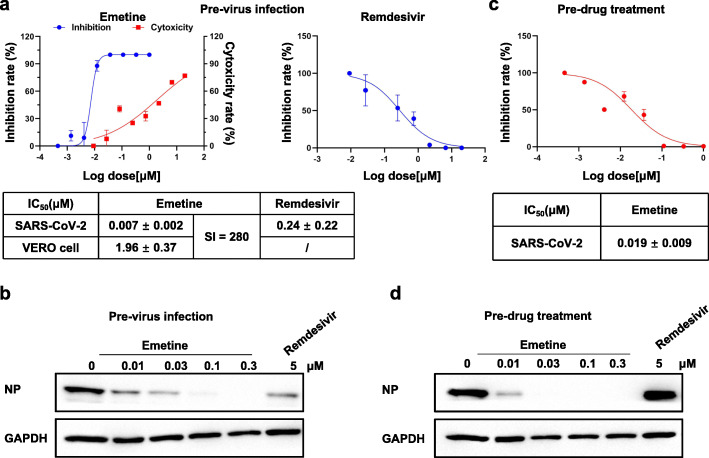
Antiviral effect of Emetine in Vero cells. **a** Antiviral replication effect of Emetine in Vero cells (EC_50_). Cells were pretreated with virus (30 TCID50) in 96 well plates for 1.5 h then virus was washed away and increasing concentrations of drugs (Emetine, Remdesivir) were added and remained for 48 h before qRT-PCR analysis. Cytotoxicity of Emetine against Vero cells were tested with CCK8 assay. **b** Western blot analysis of nucleocapsid level in Vero cells for anti-replication effect. Cells were infected by virus (30 TCID 50) in 6-well plate for 1.5 h then washed way and increasing concentrations of drugs were added and remained for 24 h until analysis. **c** Antiviral entry effect of Emetine in Vero cells. Cells in the 96-well plate were pretreated with increasing concentrations of Emetine for 3.5 h before virus (30 TCID50) was added and followed by co-incubation for 1.5 h. Then both the drug and virus were washed away and cells remained in the maintenance medium for 24 h before PCR analysis. **d** Western-blot analysis of nucleocapsid level in Vero cells for anti-entry effect. Cells were pretreated with increasing concentrations of drugs for 3.5 h in 6-well plate before virus (30 TCID50) was added and followed by co-incubation for 1.5 h. Then both the drug and virus were washed away and cells remained in the maintenance medium for 24 h before analysis

We first examined antiviral replication effect of Emetine in Vero cells. In the Pre-virus-infected Vero cells (with 30 TCID50), Emetine displayed a potent antiviral replication effect with an EC_50_ of 0.007 μM, which was over 30-fold more effective than in parallel tested Remdesivir (EC_50_: 0.24 μM). Meanwhile Emetine exhibited a selectivity index (SI) of 280 in the cytotoxicity assay with Vero cells (CC_50_: 1.96 μM) (Fig. 1a). Western blot analysis showed that the SARS-CoV-2 specific nucleocapsid level was dose-dependently decreased by Emetine, which encapsulated the results observed from qRT-PCR based virus replication inhibition assay (Fig. 1b). In addition, we also investigated the virus entry blocking effect of Emetine in Vero cells. The results demonstrated that pre-drug treatment for 3.5 h could dose-dependently block the virus entry with an EC_50_ of 0.019 μM (Fig. 1c). Western blot analysis of SARS-CoV-2 specific nucleocapsid level also confirmed this trend (Fig. 1d). Interestingly, although Remdesivir could potently inhibited the virus replication in cell, but it could not block the virus entry, which was consistent with the previous report [13].

### In vitro anti-inflamatory effects of emetine

**Fig. 2:**
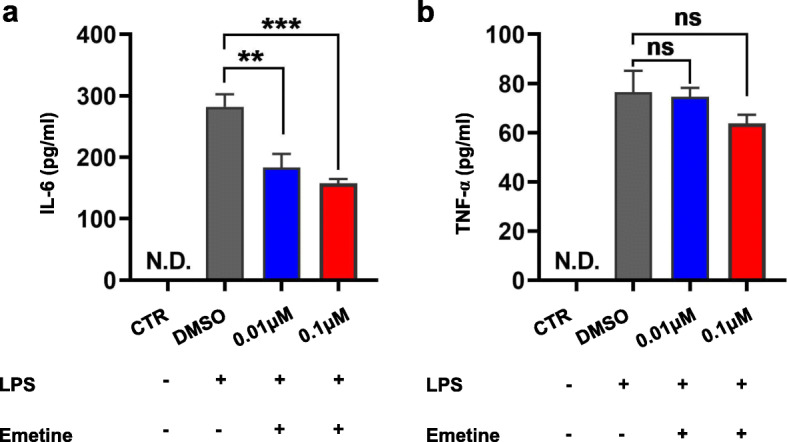
Anti-inflamatory effect of Emetine. **a** Quantification of protein levels of IL-6 and **b** TNF-α in cultural supernatant from M1 macrophages after treatment with emetine or vehicle for 36 h. Results are expressed as means ±S.D. (*n* = 3). NS: not significantly different. ** or ***: significantly different from the corresponding control (Ctrl) respectively with *p* < 0.01 or 0.001

Since virus infection induced elevated expression level of inflammatory factors such as IL-6 and TNF-α have been reported to be associated with severity of the disease, we next examined the anti-inflamatory effect of Emetine in the M1-polarized THP-1 macrophages [14]. The results showed that 0.01 μM of Emetine could significantly reduce the LPS induced IL-6 expression level (Fig. 2a). Meanwhile, Emetine exhibited moderate effect for the cytokine TNF-α epression at 0.1 μM (Fig. 2b).

### In vivo pharmacokinetic study of emetine

In order to obtain an envision for the clinical translation, we then tried to examine the pharmacokinetic (PK) profiles of Emetine in the preclinical in vivo models. Although there has been some random PK studies either with Emetine itself or Ipecac syrup (containing Emetine) [15, 16], in order to obtain the detailed PK spectrum, we first systematically investigated Emetine in different species including mice, rats and beagle dogs to depict an overall picture (Table 1). The results showed that in mice and rats, oral administration of Emetine reached maximum concentration (C_max_) in the blood plasma in a relatively long time (T_max_: 1.67 h and 9 h for the mice and rats respectively). It was absorbed much faster in dogs (T_max_: 0.25 h). In addition, across all three species, both the C_max_ and drug exposure (AUC) in the blood plasma were relatively low. However, the tissues distribution (V_z_) was very high, which indicated that Emetine might be largely enriched in the tissues after absorbtion but not remained in the plasma. The half-life(T_1/2_) in the blood plasma across all the three species were relatively long, which indicated that the drug was metabolized slowly in vivo.

**Table 1:** In vivo pharmacokinetic parameters of emetine in mice, rats and beagle dogs (*n* = 3)

Parameters	Units	Mice		Rat		Dog	
		I.V.	P.O.	I.V.	P.O.	I.V.	P.O.
		(1 mg/kg)	(10 mg/kg)	(1 mg/kg)	(10 mg/kg)	(1 mg/kg)	(5 mg/kg)
T_1/2_	hr	6.24 ± 1.17	15.95 ± 8.22	4.46 ± 1.18	5.16 ± 4.16	2.45 ± 0.91	7.99 ± 0.35
T_max_	hr	0.03 ± 0.00	1.67 ± 0.58	0.03 ± 0.00	9.00 ± 0.00	0.03 ± 0.00	0.25 ± 0.00
C_max_	ng/mL	110.7 ± 13.5	61.6 ± 15.1	26.9 ± 4.8	12.2 ± 3.3	260.0 ± 66.7	244.9 ± 164.4
C_0_	ng/mL	172 ± 86	–	33.5 ± 2.4	–	351 ± 44	–
AUC_0-t_	hr*ng/mL	120 ± 26	811 ± 208	29.8 ± 6.1	172 ± 18	449 ± 298	1547 ± 964
AUC_0-∞_	hr*ng/mL	258 ± 71	1343 ± 625	39.8 ± 11.3	220 ± 28	468 ± 298	1736 ± 1080
Vz	mL/kg	38,430 ± 19,651	174,470 ± 45,790	162,918 ± 11,043	320,304 ± 216,100	9548 ± 5261	51,033 ± 45,021
Cl	mL/hr./kg	4118 ± 1335	8611 ± 3893	26,707 ± 8401	45,918 ± 5515	2749 ± 1517	4377 ± 3748
AUMC_0-t_	hr*hr.*ng/mL	616 ± 181	8355 ± 2710	131 ± 18	1989 ± 15	1331 ± 695	12,464 ± 9313
AUMC_0-∞_	hr*hr.*ng/mL	3472 ± 1149	36,666 ± 31,176	320 ± 141	3676 ± 1871	1626 ± 679	19,162 ± 13,435
MRT_0-t_	hr	5.06 ± 0.57	10.17 ± 1.31	4.41 ± 0.31	11.63 ± 1.33	3.20 ± 0.78	7.63 ± 1.32
MRT_0-∞_	hr	13.36 ± 1.37	24.26 ± 10.01	7.76 ± 1.81	16.20 ± 6.00	3.82 ± 0.86	10.72 ± 1.21
F (%)			52.1 ± 24.2		55.3 ± 7.1		74.2 ± 46.2

**Fig. 3:**
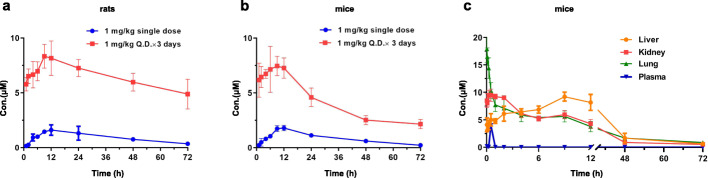
Mean lung concentration–time curves of Emetine in rats and mice after oral administration at a single dose of 1 mg/kg and multi-doses of Q.D. 1 mg/kg in three consecutive days (*n* = 3). **a** Distribution of Emetine in the lung of rats post 72 h with 1 mg/kg single oral dose and 1 mg/kg Q.D. for 3 days. **b** Distribution of Emetine in the lung of mice post 72 h with 1 mg/kg single oral dose and 1 mg/kg Q.D. for 3 days. **c** Distribution of Emetine in different tissues of mice after intravenous administration of 1 mg/kg in mice

The long half-life and potential high tissue distribution encouraged us to evaluate Emetine in the rats with 1 mg/kg single dose (P.O.) and collected the data up to 72 h (Fig. 3a; Supplementary Table 1). Interestingly, the drug exhibited high concentrations in the lung in a time dependent manner. At 12 h (T_max_) the concentration reached to maximum of 1.61 μM and then gradually decreased to 0.36 μM at 72 h which was still much higher than the EC_50_ (7 nM) for inhibition of virus replication. This was in accordance with the previous study with Ipecac syrup that Emetine could be enriched in the tissues such as lung [16], which was especially important for the COVID-19 since the lung was the major target tissue of the SARS-CoV-2 virus. In mice a similar trend was observed and at 12 h the maxium concentration (1.8 μM) was reached in the lung and gradually decreased to 0.23 μM which was still 30-fold higher than the EC_50_ for the inhibition of virus replication (Fig. 3b; Supplementary Table 1).

Inspired by this data, we then performed continuous oral administration for 3 days (1 mg/kg/day) for both mice and rats (Fig. 3a, b; Supplementary Table 1). The data showed that after stopped the drug dose there is a heavy drug accumulation in the lung. At 1 h post drug dosage Emetine was enriched to 5–6 μM which was over 30-fold than the single dosage both in rats and mice. Post 9 h both in rats and mice Emetine reached to maximum concentration (C_max_) which was about 4–5-fold higher than single dose at the same time point. Even 72 h of post drug administration Emetine was still remained high concentration in the lung of rats (4.89 μM) and mice (2.17 μM), which was still over 30 to 70-fold higher than the EC_50_ of inhibition of virus replication (4.89 μM and 2.17 μM Vs 7 nM).

Evidence has shown that human ACE2, the main host cell receptor of the SARS-CoV-2 virus for the entry, has high expression in lung, kidney and liver etc. tissues [17]. Therefore, we then expanded detection of distribution of Emetine in those tissues (Table 2). Given the fact that Emetine containing drugs were available both as oral and I.V. injection formula, this time we performed 1 mg/kg single dose I. V. injection in mice. The results showed that Emetine quickly reached to maximum concentration (Cmax: lung (8582 ng/mL) > kidney (4552 ng/mL) > liver (4425 ng/mL) in the lung and kidney but slowly in liver after injection (Tmax: lung (0.03 h) > kidney (0.5 h) > Liver (9 h)). It was worthy to note that Emetine had a similar T_max_ of lung and blood plasma which indicated that after administration it would quickly reach to the lung. Furthermore, in comparison, the high distribution concentrations in these three tissues could retain for 12 h and were much higher than them in the blood plasma (Fig. 3c; Supplementary Table 2).

**Table 2:** Pharmacokinetic parameters of Emetine in liver, kidney, lung, plasma after intravenous administration of 1 mg/kg Emetine in mice (*n* = 3)^a^

Parameters	Units	Liver	Kidney	Lung
T_1/2_	hr	17.31	20.89	11.48
T_max_	hr	9.00	0.50	0.03
C_max_	ng/mL	4425	4552	8582
AUC_0-t_	hr*ng/mL	41,289	36,959	35,381
AUC_0-∞_	hr*ng/mL	139,259	99,074	65,395
Vz	mL/kg	179	304	253
Cl	mL/hr./kg	7.18	10.1	15.3
AUMC_0-t_	hr*hr.*ng/mL	275,511	193,238	181,502
AUMC_0-∞_	hr*hr.*ng/mL	3,897,180	2,810,677	1,038,814
MRT_0-t_	hr	6.67	5.23	5.13
MRT_0-∞_	hr	27.99	28.37	15.89

^a^Pharmacokinetic parameters are calculated from the average value of tissue drug concentration-time curve

## Discussion

The unexpected worldwide outbreak of COVID-19 caused by a new coronavirus SARS-CoV-2 infection has emerged as a global public health crisis. Besides the effective social controlling strategy such as quarantine, social distancing, proper individual protection, as well as the identification of the infections by efficient diagnosis, effective antiviral therapies are critical and highly urgent for the infection treatment and control. In the very limited time course it is very difficult to develop new drugs for a new disease. Therefore, “old” drug repurposing is the most efficient and practical approach to identify the clinically available therapy for the urgent demand due to their safety profiles being well documented. Recently the “old” drugs chloroquine and hydroxychloroquine, usually used as anti-malaria or immunosuppressing reagent, have been successfully reapplied to anti-SARS-CoV-2 clinical investigation and some promising primary results have been reported [13, 18]. Remdesivir, which was originally developed as an anti-Ebola virus agent, as well as a number of anti-HIV, MERS-CoV agents were also reapplied to anti-SARS-CoV-2 for urgent clinical investigation and treatments [19, 20].

Emetine, a natural plant alkaloid found in the ipecac root, has a long medical use history as a vomiting inducing reagent for the acute detoxification in the form of Ipecac syrup mixture by oral administration of relatively large dose. Low dose of Ipecac syrup itself or combined with other herbals have been widely used as anti-cough therapies for decades in Asia. In addition, pure chemical form of Emetine was also developed as an anti-protozoal therapy by subcutaneous injection. Furthermore, Emetine has been reported to display broad spectrum of antiviral activities including the two highly pathogenic coronavirus SARS-CoV and MERS-CoV in vitro [21]. Emetine has also exhibited potent inhibitory activity against human enterovirus and cytomegalovirus both in vitro and in vivo animal models at the very low dosage [22, 23]. The primary mechanistic study showed that Emetine might exert its antiviral effect through blocking the virus associated translation but the detailed mechanism still requires further exploration.

The clinical use of Emetine as anti-protozoal drug was 1 mg/kg/day (60 kg human weight) and no more than 60 mg/day as the total dosage by subcutaneous injection. Regarding to the safety profiles of Emetine, a phase I clinical study showed that 15/35 patients observed injection site pain and most of patients suffer muscle weakness with 1 mg/kg subcutaneous injection [24]. While another phase I clinical study with 2.7–10.4 mg/kg intravenous injection of Emetine did not observe the muscle weakness [25]. No serious side effect has been observed with the 1 mg/kg injection for the human use in the clinic. The most tolerated dosage is about 10 mg/kg for the human being. Both US Pharmacopoeia and the earlier edition before 2013 of the British Pharmacopoeia have listed injection formulation of Emetine as reference standard [11]. As an anti-cough agent Emetine was often used as Ipecac syrup mixure together with other herbs at very lose dose. In China a widely used TCM called XIAOERHUATANZHIKEKELI has been applied to children under 10 years old as OTC drug for general cough relasing, which contained 0.4–0.5 mg Emetine/bag. With 2 bags/ each time and 3 times a day dosage no serious side effects were reported except rare vomettings.

**Fig. 4:**
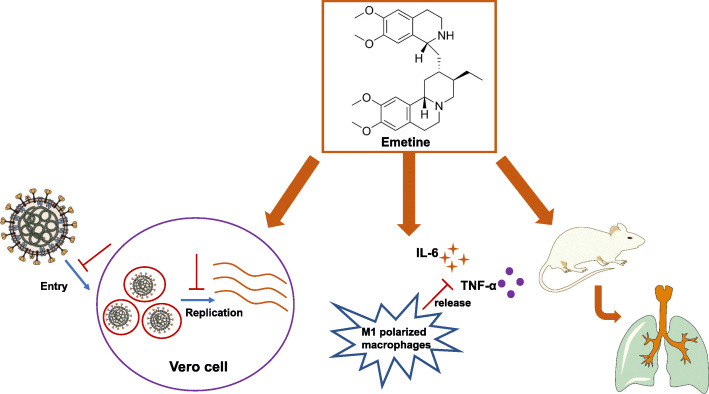
Graphic illustration of action Model for Emetine as Anti-SARS-CoV-2 Virus Therapy. Firstly, Emetine could potently inhibit the SARS-CoV-2 virus replication and displayed virus entry blocking effect in Vero cells at low dose. Secondly, Emetine could significantly reduce the LPS induced IL-6 protein level and moderately reduce the TNF-α protein level in the M1 polarized THP-1 macrophages. In addition, in vivo animal PK study revealed that Emetine was enriched in the lung tissue and had a long retention time. In summary, the potent in vitro antiviral replication efficacy, the high enrichment in target tissue combining with the well documented safety profiles in human indicated that low dose of Emetine might be a potentially effective anti-SARS-CoV-2 infection therapy

In present study we found that Emetine could potently inhibit the SARS-CoV-2 virus replication in the Vero cells (EC_50_: 7 nM), which is similar to the inhibitory activity against SARS-CoV and MERS-CoV virus as previously reported [9]. During the preparation of this manuscript, Yen et al. also found that Emetine was potent agasint SARS-CoV-2 virus in a HTS screening [12]. In addition, we also found that at the EC_50_ concentration of virus replication inhibition, Emetine could effectively reduce the expression level of IL-6, which has been reported to be elevated in the sever patients. This anti-inflamatory effect indicates that it might be potentially useful for combating the cytokine storms, which has been observed in many ICU admitted patients [26]. One advantage of Emetine is its high enrichment in the SARS-CoV-2 targeted tissues including the lung, kidney and liver. Especially it could quickly reach high concentration in the lung with either oral or I.V. injection (summary model, Fig. 4). Most importantly, Emetine could retain the high concentration in the lung tissues over 12 h at the dose of 1 mg/kg either with oral or I.V. injection. 1 mg/kg dose in the mice could provide 1.8 μM (oral) and 3.8 μM (I.V.) concentration in the lung, which is about 250-fold and 500-fold of EC_50_ for the virus replication inhibition.

Finally, it is worthy to note that although not included in the present study due to the resource limitation, it would be great to further elucidate detailed antivirus mechanism and perform in vivo efficacy study with the human ACE2 gene engineered animal models, which would help to complete the evidence chains for the clinical translation of the Emetine as an anti-COVID-19 therapy.

## Materials and methods

### Chemicals

Emetine, Remdesivir were purchased from Haoyuan Chemexpress Inc. (Shanghai, P.R.China).

### Antibodies and immunoblotting

SARS-CoV-2/2019-nCoV Nucleocapsid Antibody was purchased from Sino biological (Beijing, P.R.China, Cat: 40588-T62), GAPDH antibody was purchased from Cell Signaling Technology (Danvers, MA, Cat: D16H11).

### Cell lines and virus

The African green monkey kidney (Vero) cells was obtained from ATCC and cultured in Dulbecco’s Modified Eagle’s media (Corning, USA) with 10% fetal bovine serum (FBS) and supplemented with 2% L-glutamine 1% penicillin/streptomycin at 37 °C with 5% CO_2_. Patient-derived COVID-19 (SZ005) was isolated by the Anhui Provincial Center for Disease Control and Prevention. The viral titer was determined by 50% tissue culture infective dose (TCID50) according to the cytopathic effect by use of karber method. All the infection experiments were performed in a biosafety level-3 (BSL-3) laboratory.

### In vitro antiviral activities efficacy and cytotoxicity in Vero cells

Vero cells were seeded on 96 well plates with a density of 1 × 10^5^ cells/well and infected with 30 TCID50 virus at 37 °C. After incubation for 60 min, cells were washed and serial concentrations of compounds were added to cells. After 48 h of incubation at 37 °C, the cell culture were subjected to a viral load assay by qRT-PCR. Monolayers of Vero cells were grown in 96-well culture plates. The compounds of various concentrations were added into the plates. Cell proliferation was determined after treatment with compounds for 48 h. The cytotoxic effects on Vero cells was evaluated by CCK-8 assay (MedChemExpress, China) according to the manufacturer’s instructions, and absorbance was measured using an iMark microplate reader (Bio-Red) at 450 nm. Data were normalized to the control groups (DMSO) and are presented as the mean of three independent measurements with standard error < 10%. The GI_50_ values were calculated using GraphPad Prism 7.0.

### Detection of anti-SARS-CoV-2 efficacy and reverse transcriptase-quantitative PCR analysis (qRT-PCR)

After viral infection and drug treatment for 48 h, the cell culture was subjected to multiple freeze/thaw cycles for cell lysis and the lysates were used for viral RNA isolation on an automatic nucleic acid extraction workstation according to manufacturer instructions. Reverse transcription was performed with TaqMan® Fast Virus 1-Step Master Mix (Thermo fisher, Catalog Numbers 4,444,432). Briefly, 2 μL viral RNA were used as template for one-step quantitative PCR. The full-length S gene of SARS-CoV-2 was synthesized and cloned into pcDNA3.1 as positive control plasmid. A dilution series of positive control (10–10,000,000 copies per μL) were used to establish a standard curve for determining the initial starting amount of the target template in experimental samples.

The primes and probe used for quantitative PCR:

- SB-F: GGCTGTTTAATAGGGGCTGAAC
- SB-R: ACCAAGTGACATAGTGTAGGCA
- SB probe: 5′ FAM-AGACTAATTCTCCTCGGCGGGCACG-BHQ

### Western blot analysis

For “Pre-virus infection” treatment, Vero cells were seeded on 6 well plates with a density of 5 × 10^5^ cells/well and infected with 30 TCID50 virus at 37 °C. After incubation for 1.5 h, cells were washed and serial concentrations of compounds was added to cells. Twenty-four hours later, the cells were collected for western blot analysis. For “Pre-drug treatment” treatment, Vero cells were pre-treated with serial concentrations of compounds for 3.5 h, and the 30 TCID50 virus was subsequently added to allow infection for 1.5 h. Then, the virus-drug mixture was removed and cells were further cultured with fresh drug-containing medium. Twenty-four hours later, the cells were collected for western blot analysis. NP antibody and GAPDH antibodies were used for immunoblotting.

### Inflammatory cytokine expression analysis

Human monocytic THP-1 cells were maintained in RPMI 1640 medium (Corning, USA) supplemented with 2% L-glutamine,10% fetal bovine serum (FBS), 1% penicillin/ streptomycin and 50 pM ß-mercaptoethanol. THP-1 monocytes are differentiated into macrophages by 24 h incubation with 150 nM phorbol 12-myristate 13-acetate (PMA, Sigma, P8139) followed by 24 h incubation in RPMI medium. Macrophages were polarized in M1 macrophages by incubation with 20 ng/ml of IFN-γ (R&D system, #285-IF) and 100 ng/ml of LPS (Sigma, #8630). Next, they were incubated in 5% CO_2_ atmosphere at 37 °C supplemented with various concentrations of Emetine for 36 h. Cytokine secretion in the culture medium was assayed using an ELISA kit according to the procedure recommended by the supplier (IL-6 (MultiSciences, 70-EK106/2–96), TNFα (MultiSciences,70-EK182HS-96)).

### Animal experimentation

The study protocol was approved by the Animal Ethics Committee of Hefei Institutes of Physical Science, Chinese Academy of Sciences (Hefei, China). The male mice (20–25 g) and Sprague-Dawley rats (180–220 g) were provided by Laboratory Animal Center of Anhui Medical University (Hefei, China), and the beagle dogs were obtained from Nanjing Bosgene Biotech Co., Ltd. All the animals were housed under control conditions (temperature 23 ± 2 °C, humidity 50% ± 5%, 12 h dark/light cycle) with standardized diet and acclimatized for 7 days, prior to the experiments. All the animals were fasted 12 h before the administration of emetine but with free access to water.

### Pharmacokinetics study

The 12 mice, rats, or dogs were randomly and equally divided into two groups for pharmacokinetics and bioavailability study. One group received a single intravenous administration of Emetine at 1 mg/kg, and the other group was treated by oral administration of emetine solution at a dose of 10 mg/kg for mice or rats, but 5 mg/kg for dogs. After dosage, blood samples were collected into heparinized tubes at 2, 5, 15, 30, 60, 120, 240, 360, 540, and 720 min for the i.v. group, and at 5, 15, 30, 60, 90, 120, 240, 360, 540, 720 and 1440 min for the i.g. group. Plasma (100 μL) was isolated from the blood sample by centrifuging at 4 °C and 6000 rpm for 3 min and then stored at − 80 °C until analysis. An aliquot of 100 μL of each plasma sample was mixed with 20 μL of internal standard working solution (200 ng/mL of caffeine). Methanol (400 μL) was then added for precipitation. After vortexing for 5 min and centrifuging at 14000 rpm for 10 min, 5 μL of the supernatant was injected for LC-MS/MS analysis.

### Tissue distribution study

Seventy-two mice or rats were randomly and equally divided into two groups. One group was p.o. administered a single dose of 1 mg/kg Emetine, and the other proup was p.o. administered at a dose of 1 mg/kg Emetine q.d. for three consecutive days. Tissues (liver, lung, and kidney) were promptly harvested at 1, 2, 4, 6, 9, 12, 24, 48, 72, and 144 h. Each tissue sample was homogenized (Tissue sample:saline ratio of 1:5, w/v). Tissue samples were stored at − 80 °C until further analysis. The preparation process for analysis was the same as described above for plasma.

### LC-MS/MS analysis

The LC-MS/MS system consisted of a SHIMADZU LC-30 AD series HPLC system (Shimadzu, Kyoto, Japan) and API4000 triple quadrupole mass spectrometer (Applied Biosystems Sciex, Ont., Canada) with electrospray ionization (ESI) source. Separation was carried out on a C18 column (2.1 × 100 mm, 5 μm, Hanbon, Jiangsu, China) kept at 40 °C. The mobile phase consisted of water (containing 0.1% formic acid, mobile phase A) and methanol (mobile phase B) with rate set at 0.3 mL/min. The optimal gradient elution curve included 0–1.0 min, 10% B; a linear increase to 90% a within 0.5 min; 90% B for 3.5 min; a linear decrease to 10% B within 0.5 min. 10% B for 1.5 min; 7.00 min, stop.

LC-MS/MS was performed in the positive and multiple reaction monitoring (MRM) mode. Detected ions were at m/z 481.2 → 246.0 for emetine and m/z 195.0 → 138.0 for the internal standard (caffeine). The parameters of mass spectrometer were optimized as follows: curtain gas, gas1 and gas2 were 25, 45 and 45 psi respectively; dwell time was 200 ms; source temperature was 500 °C; ion spray voltage was 5500 V. Compound parameters including declustering potential, collision energy, entrance potential and collision exit potential were optimized at 110, 44, 10 and 13.5 V for emetine and 76, 28, 10 and 10 V for internal standard, respectively.

### Pharmacokinetic and statistical analysis

The PK parameters were analyzed through the noncompartment model using WinNonlin 6.1 software (Pharsight Corporation, Mountain View, USA), including half-life (T_1/2_), the peak of the plasma concentration (C_max_), the time to peak of the plasma concentration (T_max_), the area under the plasma concentration-time curve during the period of observation (AUC_0-t_), the area under the plasma concentration-time curve from zero to infinity (AUC_0-∞_), clearance (CL), apparent volume of distribution (V_d_), and the mean residence time. The oral bioavailability (F) is calculated according to the following equation: F = AUC_0-∞_ (oral)/AUC_0-∞_ (iv) × dose (iv)/dose (oral) × 100%.

## Supplementary Information

**Additional file 1: Table S1.** Distribution of Emetine in lungs of rats and mice after oral administration at a single dose of 1 mg/kg and multi-doses of Q.D. 1 mg/kg in three consecutive days (*n* = 3). **Table S2.** Distribution of Emetine in different tissues of mice after intravenous administration of 1 mg/kg in mice (*n* = 3).

## Data Availability

Not applicable.

## References

[1] Yuen KS, Ye ZW, Fung SY, Chan CP, Jin DY (2020). SARS-CoV-2 and COVID-19: the most important research questions. Cell Biosci.

[2] Lu RJ, Zhao X, Li J, Niu PH, Yang B, Wu HL (2020). Genomic characterisation and epidemiology of 2019 novel coronavirus: implications for virus origins and receptor binding. Lancet.

[3] Zhu N, Zhang D, Wang W, Li X, Yang B, Song J (2020). A novel coronavirus from patients with pneumonia in China, 2019. N Engl J Med.

[4] Jiang SB, Du LY, Shi ZL (2020). An emerging coronavirus causing pneumonia outbreak in Wuhan, China: calling for developing therapeutic and prophylactic strategies. Emerg Microbes Infect.

[5] Wan S, Yi Q, Fan S, Lv J, Zhang X, Guo L, et al. Characteristics of lymphocyte subsets and cytokines in peripheral blood of 123 hospitalized 3 patients with 2019 novel coronavirus pneumonia (NCP). medRxiv. 2020. 10.1101/2020.02.10.20021832.

[6] Guan WJ, Ni ZY, Hu Y, Liang WH, Ou CQ, He JX, et al. Clinical characteristics of coronavirus disease 2019 in China. N Engl J Med. 2020. 10.1056/NEJMoa2002032. 10.1056/NEJMoa2002032 PMC7092819 32109013

[7] Chaves Valadao AL, Abreu CM, Dias JZ, Arantes P, Verli H, Tanuri A (2015). Natural plant alkaloid (emetine) inhibits HIV-1 replication by interfering with reverse transcriptase activity. Molecules.

[8] Yang S, Xu M, Lee EM, Gorshkov K, Shiryaev SA, He S (2018). Emetine inhibits Zika and Ebola virus infections through two molecular mechanisms: inhibiting viral replication and decreasing viral entry. Cell Discov.

[9] Dyall J, Coleman CM, Hart BJ, Venkataraman T, Holbrook MR, Kindrachuk J (2014). Repurposing of clinically developed drugs for treatment of Middle East respiratory syndrome coronavirus infection. Antimicrob Agents Chemother.

[10] Shen L, Niu J, Wang C, Huang B, Wang W, Zhu N, et al. High-throughput screening and identification of potent broad-spectrum inhibitors of coronaviruses. J Virol. 2019;93. 10.1128/JVI.00023-19. 10.1128/JVI.00023-19 PMC6613765 30918074

[11] Bleasel MD, Peterson GM. Emetine, ipecac, ipecac alkaloids and analogues as potential antiviral agents for coronaviruses. Pharmaceuticals (Basel). 2020;13. 10.3390/ph13030051. 10.3390/ph13030051 PMC7151655 32245264

[12] Choy KT, Wong AY, Kaewpreedee P, Sia SF, Chen D, Hui KPY (2020). Remdesivir, lopinavir, emetine, and homoharringtonine inhibit SARS-CoV-2 replication in vitro. Antivir Res.

[13] Wang ML, Cao RY, Zhang LK, Yang XL, Liu J, Xu MY (2020). Remdesivir and chloroquine effectively inhibit the recently emerged novel coronavirus (2019-nCoV) in vitro. Cell Res.

[14] Chanput W, Mes JJ, Savelkoul HFJ, Wichers HJ (2013). Characterization of polarized THP-1 macrophages and polarizing ability of LPS and food compounds. Food Funct.

[15] Asano T, Watanabe J, Sadakane C, Ishihara K, Hirakura K, Wakui Y (2002). Biotransformation of the ipecac alkaloids cephaeline and emetine from ipecac syrup in rats. Eur J Drug Metab Pharmacokinet.

[16] Asano T, Ishihara K, Wakui Y, Yanagisawa T, Kimura M, Kamei H (2002). Absorption, distribution and excretion of H-3-labeled cephaeline- and emetine-spiked ipecac syrup in rats. Eur J Drug Metab Ph.

[17] Xu H, Zhong L, Deng JX, Peng JK, Dan HX, Zeng X, et al. High expression of ACE2 receptor of 2019-nCoV on the epithelial cells of oral mucosa. Int J Oral Sci. 2020;12. 10.1038/s41368-020-0074-x. 10.1038/s41368-020-0074-x PMC7039956 32094336

[18] Liu J, Cao RY, Xu MY, Wang X, Zhang HY, Hu HR, et al. Hydroxychloroquine, a less toxic derivative of chloroquine, is effective in inhibiting SARS-CoV-2 infection in vitro. Cell Discov. 2020;6. 10.1038/s41421-020-0156-0. 10.1038/s41421-020-0156-0 PMC7078228 32194981

[19] Sims AC, Tilton SC, Menachery VD, Gralinski LE, Schafer A, Matzke MM (2013). Release of severe acute respiratory syndrome coronavirus nuclear import block enhances host transcription in human lung cells. J Virol.

[20] Sheahan TP, Sims AC, Graham RL, Menachery VD, Gralinski LE, Case JB, et al. Broad-spectrum antiviral GS-5734 inhibits both epidemic and zoonotic coronaviruses. Sci Transl Med. 2017;9. 10.1126/scitranslmed.aal3653. 10.1126/scitranslmed.aal3653 PMC5567817 28659436

[21] Andersen PI, Ianevski A, Lysvand H, Vitkauskiene A, Oksenych V, Bjoras M (2020). Discovery and development of safe-in-man broad-spectrum antiviral agents. Int J Infect Dis.

[22] Mukhopadhyay R, Roy S, Venkatadri R, Su YP, Ye WJ, Barnaeva E, et al. Efficacy and mechanism of action of low dose emetine against human cytomegalovirus. PLoS Pathog. 2016;12. 10.1371/journal.ppat.1005717. 10.1371/journal.ppat.1005717 PMC4919066 27336364

[23] Tang Q, Li SL, Du LQ, Chen SH, Gao JY, Cai Y, et al. Emetine protects mice from enterovirus infection by inhibiting viral translation. Antivir Res. 2020;173. 10.1016/j.antiviral.2019.104650. 10.1016/j.antiviral.2019.104650 31734270

[24] Mastrangelo MJ, Grage TB, Bellet RE, Weiss AJ (1973). A phase I study of emetine hydrochloride (NSC 33669) in solid tumors. Cancer.

[25] Panettie F, Coltman CA (1971). Phase-I experience with emetine hydrochloride (Nsc-33669) as an antitumor agent. Cancer.

[26] Huang C, Wang Y, Li X (2020). Clinical features of patients infected with 2019 novel coronavirus in Wuhan, China (vol 395, pg 497, 2020). Lancet.

