# Supplementary material for: Low dose of emetine as potential anti-SARS-CoV-2 virus therapy: preclinical in vitro inhibition and in vivo pharmacokinetic evidences
Source: Mol Biomed. 2020 Nov 30;1:14. doi: 10.1186/s43556-020-00018-9 (PMC7700809; doi:10.1186/s43556-020-00018-9)
Supplement: Supplementary file 1 — Additional file 1: Table S1. Distribution of Emetine in lungs of rats and mice after oral administration at a single dose of 1 mg/kg and multi-doses of Q.D. 1 mg/kg in three consecutive days (n = 3). Table S2. Distribution of Emetine in different tissues of mice after intravenous administration of 1 mg/kg in mice (n = 3). [file 43556_2020_18_MOESM1_ESM.docx]

**Supplementary Materials for**

**Table s1: Distribution of Emetine** **in lungs of rats and mice after oral administration at a single dose of 1 mg/kg and multi-doses of Q.D. 1 mg/kg in three consecutive days (n=3).**

| Time (h) | Rat lung con.(ng/mL) | | Mice lung con.(ng/mL) | |
| --- | --- | --- | --- | --- |
|  | 1 mg/kg  (P.O.) | 1 mg/kg  ( Q.D.× 3 days, P.O.) | 1 mg/kg  (P.O.) | 1 mg/kg  ( Q.D.× 3 days, P.O.) |
| 1 | 0.15±0.03 | 5.78±0.61 | 0.23±0.04 | 6.17±1.54 |
| 2 | 0.26±0.06 | 6.51±0.67 | 0.50±0.35 | 6.44±0.96 |
| 4 | 0.93±0.32 | 6.65±1.21 | 0.81±0.16 | 6.73±0.40 |
| 6 | 1.01±0.10 | 6.96±0.87 | 1.06±0.09 | 7.14±2.10 |
| 9 | 1.47±0.09 | 8.34±1.09 | 1.75±0.28 | 7.46±0.89 |
| 12 | 1.61±0.48 | 8.16±1.58 | 1.80±0.25 | 7.26±0.93 |
| 24 | 1.32±0.63 | 7.25±0.79 | 1.13±0.18 | 4.61±0.84 |
| 48 | 0.76±0.11 | 5.97±0.81 | 0.61±0.09 | 2.53±0.42 |
| 72 | 0.36±0.08 | 4.89±1.35 | 0.23±0.06 | 2.17±0.42 |

**Table s2: Distribution of Emetine in different tissues of mice after intravenous administration of 1 mg/kg in mice** **(n=3).**

| Time (h) | Liver (μM) | Kidney (μM) | Lung (μM) | Plasma (μM) |
| --- | --- | --- | --- | --- |
| 0.033 | 4.11±1.25 | 8.52±1.25 | 17.9±2.51 | 0.15±0.06 |
| 0.083 | 3.05±0.23 | 8.04±0.57 | 16.5±1.59 | 0.11±0.02 |
| 0.25 | 4.06±0.86 | 9.44±1.92 | 14.4±1.89 | 0.06±0.02 |
| 0.5 | 5.01±1.13 | 9.48±1.10 | 10.3±1.45 | 0.04±0.02 |
| 1 | 4.78±0.43 | 9.24±0.44 | 7.73±1.22 | 0.03±0.006 |
| 2 | 6.12±1.37 | 9.04±0.15 | 7.12±2.30 | 0.012±0.003 |
| 4 | 6.46±0.57 | 6.16±0.77 | 5.85±1.20 | 0.007±0.001 |
| 6 | 6.87±0.65 | 5.24±0.47 | 5.42±0.13 | 0.009±0.004 |
| 9 | 9.22±0.81 | 5.96±0.76 | 5.63±1.00 | 0.008±0.004 |
| 12 | 8.17±1.46 | 4.29±0.79 | 3.77±0.96 | 0.007±0.005 |
| 48 | 1.67±0.872 | 0.879±0.278 | 1.67±0.28 | ND |
| 72 | 0.620±0.024 | 0.531±0.053 | 0.852±0.075 | ND |
| 144 | 0.129±0.041 | 0.174±0.018 | 0.263±0.034 | ND |

ND: not detected
